# Tripe palms and Malignant Acanthosis Nigricans: More than a diagnostic pointer

**DOI:** 10.1002/cnr2.1307

**Published:** 2020-10-19

**Authors:** Parmod Kumar, Megha K Mukundan, Amit Sehrawat, Deepak Sundriyal

**Affiliations:** ^1^ Department of Medical Oncology Haematology All India Institute of Medical Sciences Rishikesh India; ^2^ Department of General Medicine All India Institute of Medical Sciences Rishikesh India

**Keywords:** carcinoma ovary, malignant Acanthosis Nigricans, platinum resistance, tripe palms

## Abstract

**Background:**

Tripe palms (TP) is one of the rare cutaneous paraneoplastic manifestations of various intra‐abdominal malignancies. TP and malignant acanthosis nigricans (MAN) occur together and may precede even years before the index cancer. Though rare, the clinical significance of TP and MAN holds significance as an indicator of internal malignancy.

**Case:**

Here, we describe 71‐year, postmenopausal female with ovarian cancer who presented to us with a history of dyspepsia, abdominal distension, and weight loss. On detailed history and evaluation, it was found that she had TP and MAN 4 years before diagnosis.

**Conclusion:**

The unique presentation preceding the primary illness necessitates extensive early work‐up to look for malignancy and the initial consideration for surgery due to the tumor biology in such patients.

## 1 BACKGROUND

Tripe palms (TP), a term coined by Jacqueline Clarke, is a rare cutaneous manifestation often associated with internal malignancies.^1^ In 1993, Cohen et al published a review of all cases reported till then. The study revealed that among 87 patients of TP, 79 (91%) cases were associated with cancer. The association seen between TP and malignant acanthosis nigricans (MAN) in cancer patients was 72%.^2^ Since Cohen et al review, many case reports have consistently reported the association between malignancies involving various organs with TP and MAN. These rare dermatological findings are strong pointers of internal malignancy and warrants a detailed search for the same, imparting diagnostic significance to them.

However, to the best of our knowledge, treatment outcomes of such cases and prognostic relevance are unknown. We present a case of platinum‐refractory ovarian carcinoma, with cutaneous paraneoplastic manifestations of TP and MAN. We also propose a biologically plausible hypothesis that patients who develop TP and MAN carry an intrinsic resistance for platinum‐containing chemotherapy.

## 2 CASE PRESENTATION

A 71‐year, postmenopausal female, presented to us with a history of dyspepsia, abdominal distension, and weight loss of 2‐month duration. Earlier, she was evaluated elsewhere for similar presenting complaints and diagnosed to have adenocarcinoma carcinoma ovary (stage IIIC). She received three cycles of chemotherapy (Paclitaxel and Carboplatin), and response assessment showed a partial response, but she refused surgical options. The treating physician continued three more cycles of chemotherapy, and the patient remained on irregular follow‐up for the next three months.

After that patient presented to our institute with above mention symptoms suggestive of recurrence/progressive disease.

On further inquiry, there was a history of these skin changes for the last 4 years, but no medical help was sought. On evaluation, the patient was nondiabetic, nonhypertensive, and not suffering from any chronic medical illness.

## 3 INVESTIGATIONS

After that patient presented to our institute with above mention symptoms suggestive of recurrence/progressive disease. The patient was evaluated with ascitic fluid cytology (reported as poorly differentiated adenocarcinoma), tumor markers, and radiological imaging to confirm disease recurrence.

**FIGURE 1:**
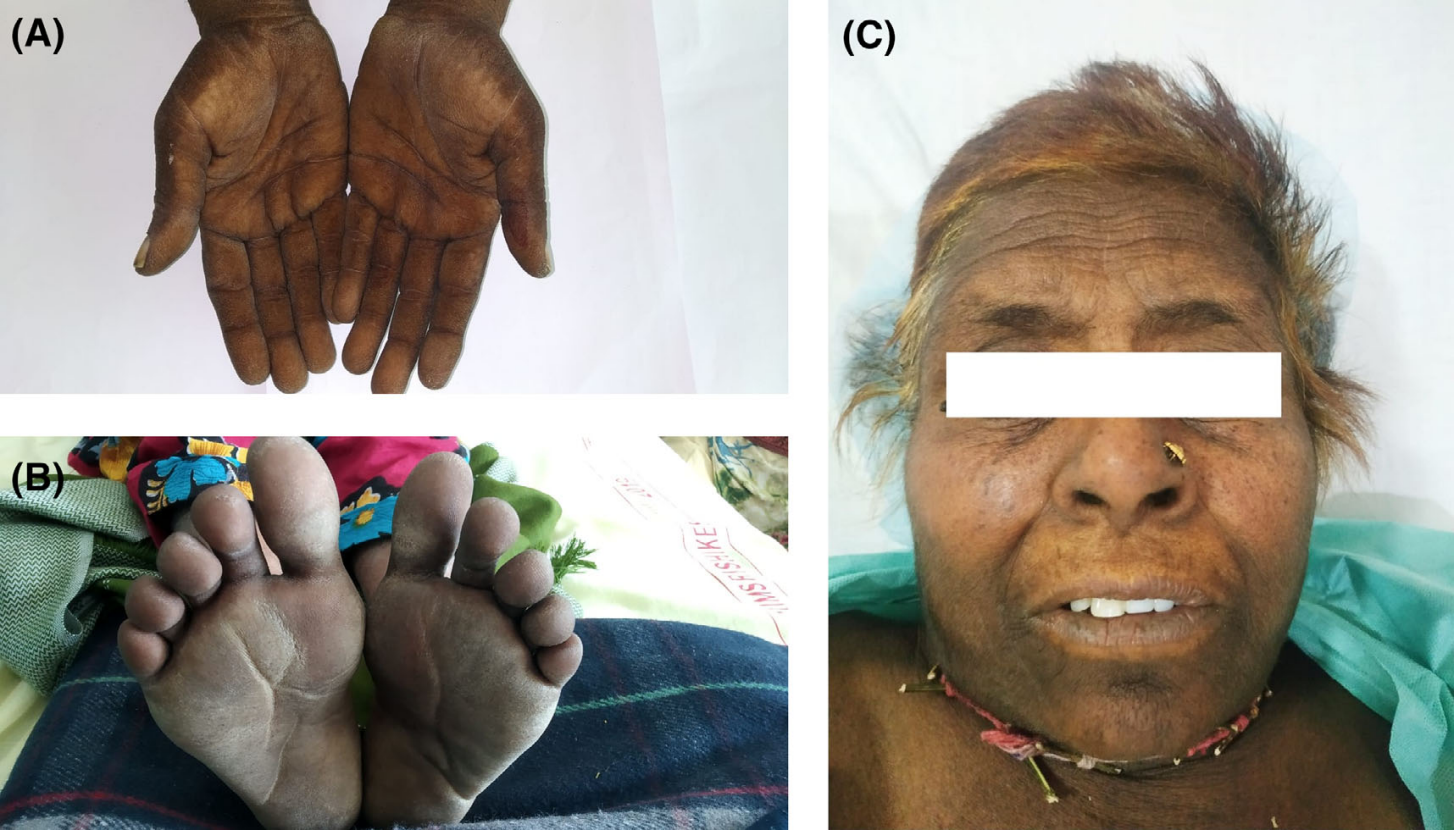
(A) Tripe palms with palmer hyperpigmentation, (B) skin changes in feet, and (C) Malignant Acanthosis Nigricans on the forehead, chin, and neck

Clinical evaluation revealed excessive thickening, course velvet‐like texture with honeycombed plaques of the palms, and soles (Figure 1A,B). Also, there was hyperpigmentation of skin folds and crease all over the body (Figure 1C).

## 4 TREATMENT AND OUTCOME

Her treatment course indicated platinum‐refractory carcinoma ovary, so second‐line Liposomal Doxorubicin chemotherapy was planned. After two cycles of chemotherapy, she developed obstructive jaundice, which was due to liver metastasis, confirmed by ultrasonography. Subsequently, the patient underwent percutaneous transhepatic biliary drainage. Her palliative plan was explained to the patient and family. As she opted for home‐based supportive care, a local hospice facility was involved for the same. The patient succumbed to her illness in May 2020.

## 5 DISCUSSION

TP and MAN are rare dermatological paraneoplastic manifestations. Their clinical relevance has been highlighted by Cohen et al, who reported that gastric and lung cancers were the most common associated primary cancers (29% and 20%, respectively). These paraneoplastic skin lesions preceded the diagnosis of cancer in 48% patient, developed simultaneously (within 1 month) in 21%, and 31% after diagnosis was established or in follow up. The representation of ovarian cancer in the study was just 3.5%.^2^

On Pubmed and Google search from 1995 to 2020, we found only five case reports of ovarian cancers with features of TP and MAN and summarized them in (Table 1). In most patients, the skin lesions were present before cancer diagnosis, and those who underwent surgical intervention had better clinical outcomes for cancer and skin lesions.

**TABLE 1:** Case reports of Carcinoma ovary presented with Tripe palm (TP) and Malignant Acanthosis Nigrican (MAN) with their treatment and response outcome

Authors	Diagnosis	Clinical presentation of skin lesion	FIGO stage	Treatment	Outcome 1.Malignancy	Follow‐up mention by author
					2. Skin lesion	
Costa et al^3^	57 years/F Adenocarcinoma	Presented for 2 years, but no evaluation	Stage IV	Palliative chemotherapy	NA	NA
Singh and Rai^4^	47 years/F Adenocarcinoma	Noticed during evaluation	Stage IIIA	Cytoreductive surgery followed by chemotherapy	NA	NA
Oh et al^5^	57 years/F Adenocarcinoma	Presenting symptom and direct search of cancer	Stage IIIc	Cytoreductive surgery followed by paclitaxel with carboplatin six cycles	CR	2 years
					Skin lesion completely recovered	
Kebria^6^	52 years/F 1.Clear cell carcinoma left ovary (Grade‐III) 2. Endometrial adenocarcinoma	Presenting symptom and direct search of cancer	Stage –IA Stage‐IA	Cytoreductive surgery followed by six cycle paclitaxel and carboplatin chemotherapy	CR	1 year
					Completely disappeared	
Low^7^	69 years/F High‐grade serous carcinoma	Presenting symptom and direct search of cancer	Stage IV	Six cycle paclitaxel and carboplatin chemotherapy followed by cytoreductive surgery	CR	2 years
					clinically Improved	

Abbreviations: CR, Complete remission; NA: information not available.

Among the patients with inoperable ovarian cancer, sensitivity to platinum‐based chemotherapy is crucial for a favorable outcome. The secretion of tumor growth factor (TGF‐α), Epidermal growth factors(EGF) by tumor cells, and upregulation of epidermal growth factor receptors‐like (EGFR, FGFR‐3) are implicated in the pathogenesis of TP and MAN.^8^ These pathways are also involved in intrinsic chemoresistance for platinum‐based chemotherapy in ovarian cancer.^9^ So, possibly “the cancer patients with TP and MAN may have intrinsic platinum resistance.” Though this is biologically plausible, it requires further evaluation.

In the current case, the patient presented in advanced‐stage ovarian cancer. Although the skin lesions of TP and MAN were there 4 years before the index malignancy, medical attention was not given in time. If TP and MAN's features were addressed and evaluated earlier, the patient might have had a better treatment course and survival. Although the initial oncological treatment plan of cytoreductive surgery following neoadjuvant chemotherapy was appropriate, consent for surgery was denied. Retrospectively, though we understand and respect the patient's decision, it might have resulted in better outcomes if it should have been pursued. To the best of our knowledge, this is the first case report of paraneoplastic TP and Man in platinum‐refractory carcinoma ovary.

### 5.1 LEARNING POINTS

- TP and MAN are rare dermatological paraneoplastic manifestations found in many cancers.
- In a large number of patients, their appearance is an essential early diagnostic pointer for detecting internal malignancies.
- Timely intervention and evaluation is the key to improve the outcomes.
- There is a biological plausibility that tumor presenting with TP and MAN may be prone for platinum‐resistance which needs to be explored further.
- Though, at present, the prognostic significance is not established; further research and awareness for this entity should be pursued.

## CONFLICT OF INTEREST

The authors have stated explicitly that there are no conflicts of interest in connection with this article.

## AUTHOR CONTRIBUTIONS

**Parmod Kumar:** Formal analysis; investigation; methodology; visualization; writing‐original draft; writing‐review and editing. **Megha Mukundan:** Conceptualization; investigation; methodology; visualization; writing‐original draft; writing‐review and editing. **Amit Sehrawat:** Conceptualization; data curation; formal analysis; investigation; methodology; resources; supervision; validation; visualization; writing‐original draft; writing‐review and editing. **Deepak Sundriyal:** Conceptualization; formal analysis; investigation; methodology; supervision; validation; visualization; writing‐original draft; writing‐review and editing.

## ETHICAL STATEMENT

No institutional ethics clearance is required for case report presentation. Written and informed consent was taken from the patient's family for publishing this report.

## Data Availability

The data that support the findings of this study are available from the corresponding author,Dr. Amit Sehrawat, upon reasonable request.

## References

[1] Clarke J . Malignant acanthosis nigricans. Clin Exp Dermatol. 1977;2:167‐170. 884896 10.1111/j.1365-2230.1977.tb01561.x

[2] Cohen PR , Grossman ME , Silvers DN , et al. Tripe palms and cancer. Clin Dermatol. 1993;11(1):165‐173. 8339193 10.1016/0738-081x(93)90114-r

[3] Costa MC , Martinez NS , Belicha MG , Leal F . Acanthosis nigricans and "tripe palm" as paraneoplastic manifestations of metastatic tumor. An Bras Dermatol. 2012;87(3):498‐500. 22714777 10.1590/s0365-05962012000300030

[4] Singh SK , Rai T . A rare case of malignant acanthosis nigricans in a lady with ovarian cancer. Indian Dermatol Online J. 2013;4(2):125‐127. 23741672 10.4103/2229-5178.110640 PMC3673379

[5] Oh CW , Yoon J , Kim CY . Malignant acanthosis nigricans associated with ovarian cancer. Case Rep Dermatol. 2010;2(2):103‐109. 20689633 10.1159/000317116 PMC2914370

[6] Kebria MM , Belinson J , Kim R , Mekhail TM . Malignant acanthosis nigricans, tripe palms and the sign of Leser‐Tre' lat, a hint to the diagnosis of early‐stage ovarian cancer: a case report and review of the literature. Gynecol Oncol. 2006;101(2):353‐355. 16443260 10.1016/j.ygyno.2005.12.024

[7] Low ZM , Gan D , Gin D . Oral Acanthosis nigricans (AN) and Leser‐Trelat sign (LT) in metastatic serous ovarian carcinoma: case report and review of literature. Open J Clin Med Cases Rep. 2017;3:1‐9.

[8] Hida Y , Kubo Y , Nishio Y , et al. Malignant acanthosis nigricans with enhanced expression of fibroblast growth factor receptor 3. Acta Derm Venereol. 2009;89(4):435‐437. 19688170 10.2340/00015555-0666

[9] Pokariyal R , Hariprasad R , Kumar L , Hariprasad G , et al. Chemotherapy resistance in advanced ovarian cancer patients. Biomark Cancer. 2019;(11):1179299X19860815. 10.1177/1179299X19860815 PMC6613062 31308780

